# A ten-year change in blood parasite infection in a sympatric wall-lizard community (genus *Podarcis*) from the Atlantic coast of Portugal

**DOI:** 10.1007/s00436-026-08693-3

**Published:** 2026-05-18

**Authors:** J. Filipe Faria, João P. Maia, D. James Harris

**Affiliations:** 1 CIBIO (Centro de Investigação Em Biodiversidade e Recursos Genéticos), Universidade do Porto, Vairão, Portugal; 2 BIOPOLIS Program in Genomics, Biodiversity and Land Planning, CIBIO, Vairão, Portugal; 3 Departamento de Biologia, Faculdade de Ciências da Universidade do Porto, Porto, Portugal; 4 Institute of Evolutionary Biology, IBE, (CSIC-Universitat Pompeu Fabra), Barcelona, Spain

**Keywords:** Endoparasite, Haemogregarine, *Karyolysus*, *Hepatozoon*, Lacertid, Climate

## Abstract

Understanding how host-parasite relationships respond to environmental factors across long periods of time is vital to understanding the dynamics of parasitic infections. We studied blood parasite infections in two sympatric species of *Podarcis* lizards in Northern Portugal between 2011 and 2013 and in 2021, analysing parasite data together with host traits and microclimatic conditions to understand how this relationship was maintained through time. Relationships between host traits and parasite infection remained consistent across years, suggesting changes to infection parameters are more dependent on environmental factors than host adaptation. Warmer and drier conditions were significantly related with higher parasite infection prevalence and drier conditions with intensity, although the magnitude of the latter was dependant on host sex. The climatic trend observed during sampled years was of cooling conditions, congruent with the decrease in parasite infection parameters observed, although the lack of intermediate time points means it is unclear if this is a trend or just an isolated drop in prevalence. We also provide insights into a framework for using data obtained through different methodologies in linear model analyses, by homogenising the varying certainty of observations from different methods.

**Supplementary Information:**

The online version contains supplementary material available at 10.1007/s00436-026-08693-3.

## Key Findings

Blood parasite infection in these host populations increase with warmer drier conditions, despite no apparent negative effect of these conditions on hosts.

Sampled years exhibit a trend of decreasing temperature, coupled with a decrease in parasite infection parameters.

Relationships between lizard host traits and parasite infection remain consistent across years, suggesting stable host-parasite relationship affected by external factors.

Proposal of a statistical framework for comparing blood parasite data from qPCR and microscopy analysis.

**Supplementary Information:**

The online version contains supplementary material available at 10.1007/s00436-026-08693-3.

## Introduction

Research has shown parasites play an indispensable role in their biological communities, influencing host population dynamics, community structure, and ecosystem functioning (Hatcher et al. 2012; Hudson et al. 2006; Poulin 1997). Despite their importance, parasites have historically been underrepresented in ecological research, particularly in long-term dynamics of infection in wild populations (Hudson et al. 2006). Understanding the long-term dynamics of host–parasite systems is essential to recognize and anticipate how both host and parasite respond to environmental changes, a knowledge made even more crucial due to the current increased climatic instability experienced worldwide. Data from different sources can be used for such research, although comparing datasets collected independently over time with differences in methodology, sampling effort, diagnostic sensitivity, identification techniques, and even reporting consistency can complicate comparisons. While certain statistical methods can be employed to overcome issues with data comparability (Anderson and Bailer 2010), they usually require prior knowledge of the specific differences between methodologies (e.g., quantified sensitivity difference), which might not be always available (Fletcher and Dixon 2012). Nevertheless, analysing this long-term data is crucial, and efforts are needed to incorporate all available data into current studies and provide more comprehensive views of these dynamic processes.

Regarding host-parasite relationships involving ectothermic hosts, whose physiological performance is strongly influenced by external temperatures, this association with climate is particularly complex (Marcogliese 2016; Poulin, 2006), as these hosts are much more dependent on external conditions to achieve the required internal body-temperature for their biological functions, including to regulate their immune system in response to parasites or other pathogens (Adolph and Porter 1993; Angilletta 2009; Woods et al. 2003). Identifying definite patterns of effect of climate on parasitism in ectothermic hosts, however, has proven challenging, as different directionality of effects have been observed across regions and taxa. Increased temperatures are often associated with an increase in parasitism in ectothermic hosts at expense of host fitness (Megía-Palma et al. 2024; Poulin and Mouritsen 2006), but the contrary has also been observed, and some authors even suggest that parasites might typically be more vulnerable to climatic changes than their hosts (Cizauskas et al. 2017). These opposing propositions may result from variations in host population dynamics, traits, and immunity, parasite thermal tolerance, and vector ecology (Arneberg et al. 1998; Morley and Lewis 2015; Song and Proctor 2020).

Lizards throughout the Western Mediterranean host apicomplexan blood parasites, from the orders Adeleorina and Eimeriorina, at varying prevalence and intensities of infection (Amo et al. 2004; Amo, Fargallo, et al. 2005; Amo, López, et al. 2005). Our study site includes two wall lizard species living in sympatry, *Podarcis bocagei* and *P. lusitanicus*, both known to host *Karyolysus* sp. (Apicomplexa: Adeleorina), sometimes referred to the genus *Hepatozoon* due to the complex taxonomy of the group (Maia et al. 2016), and *Schellackia* sp. (Apicomplexa: Eimeriorina) blood parasites (Faria et al. 2024). The most common, species of *Karyolysus*, are blood parasites with a heteroxenous life cycle, with asexual reproduction in the erythrocytes of the lizard host and sexual reproduction and sporogony in invertebrate hosts, typically hematophagous mites, which act as vectors by parasitizing the lizards and then later being ingested by them (Haklová-Kočíková et al. 2014; Reichenow 1913). Species of the genus *Schellackia* are much less prevalent and have their full life cycle in the lizard definitive host, with the hematophagous mites in this instance acting as mechanical transmitters when again parasitizing and being ingested by the lizards (Bristovetzky and Paperna 1990; Telford 2009). Regarding invertebrate ectoparasites, mites and ticks are present in the area and can be found attached to both *Podarcis* species.

The study site consists of a semi-rural area in northern Portugal along the Atlantic coast next to the town of Moledo. The habitat is a mosaic of small traditional cultivated and abandoned plots surrounded by dry-stone walls and the occasional boulder patches. It is currently separated from the sea by a paved trail and a stretch of up to 200 m of sandy dunes and rocky outcrops. The area was frequently used for the grazing of goats, although this activity has been decreasing in recent years. The landscape has also suffered anthropogenic modifications in recent years, especially in the patches closer to the town, with modern housing being built close to the beach and some of the land plots and overgrown areas being replaced by grass lawns and the dry-stone walls by cement ones. The two *Podarcis* species inhabit this area in absolute sympatry, barring microhabitat-usage differences. The more generalist *P. bocagei* can be found both all over the walled plots and in the sand dunes, while the rock-specialist *P. lusitanicus* is more restricted to the walls around plots and some boulders.

We aim to quantify and compare the prevalence and intensity of *Karyolysus* sp. parasite infection in the sympatric *P. bocagei* and *P. lusitanicus* lizards in this region, to assess how these infection parameters have changed for these two host species when exposed to the same, or very similar, factors over a 10-year period. Furthermore, we intend to understand how microclimatic conditions may be associated with temporal shifts in the dynamics of this host-parasite system.

## Materials and methods

### Study System

Sampling was conducted near Moledo, Viana do Castelo, Portugal (41.836, -8.874). A total of 636 blood samples were collected from *P. bocagei* (410) and *P. lusitanicus* (226) lizards, through the years 2011 to 2013 (534) and in 2021 (102). During 2011, sampling was conducted in April, July, and October; during 2012 and 2013 in July and October, and in 2021 in May. For each individual, sex, snout-vent length (SVL), and tail condition (as original, regenerated, or lost) were recorded, a tissue sample (tail tip) collected and preserved in 96% ethanol, and blood from the tail cut used for blood smears on microscope slides (fixed with methanol within 48 h of sampling and dyed with Giemsa stain) and stored in Whatman filter paper.

### Parasite assessment

Due to budget constraints, the way the sample analysis was performed varied from 2011 to 2013 to 2021. Originally, a quantitative PCR (qPCR) approach was used for assessing the *Karyolysus* sp. infection status and intensity in the blood samples, but in 2021 only microscopy methods were possible. To ensure that the approaches could be compared appropriately, some samples assessed using qPCR were also examined under the microscope to determine potential biases.

We extracted DNA (from the 2011–2013 samples) for qPCR analyses from blood drops stored in Whatman filter paper with a high-salt DNA extraction protocol (Sambrook et al. 1989; Maia et al. 2014). Extracted DNA was diluted in nuclease-free water (QIAGEN) to 10 ng/µl, measured in a ND-1000 Spectrophotometer (NanoDrop Technologies, Inc.). Diluted samples were amplified in a real-time polymerase chain reaction (qPCR) in a LightCycler^®^ 480 Instrument II machine (Roche), following the protocol from Maia et al. 2014 and using the primer pair JM4_F (5’-ACTCACCAGGTCCAGACATAGA-3’) and JM5_R (5’-CTCAAACTTCCTTGCGTTAGAC-3’) (Maia et al. 2014). We ran triplicates of each sample and quadruplicates of plasmid dilutions in 384 well plates, each for a total volume of 10 µl, (iQ™ SYBR^®^ Green supermix at 1x, each primer at 0.5 mM, 1 µl of 10 ng/µl genomic DNA). Using the LightCycler^®^ 480 software v1.5.0 (Roche) we exported raw qPCR results and with the algorithm implemented in LinRegPCR individually determined the baseline threshold for each plate (Ruijter et al. 2009). Using a Melting Curve Analysis (MCA; Maia et al. 2014) blood parasite identity was verified for *Karyolysus* sp. We used parasite copy numbers, as estimated by qPCR, as a measure of parasite infection intensity.

For the samples from 2021, blood smears were examined twice under 400x magnification for 5 min., with attention to the presence of parasites. Samples for which the presence of *Karyolysus* sp. blood parasites was detected (minimum of 1 parasite) were considered parasitized for parasite infection status purposes and had random areas of the blood smear photographed under 400x magnification with a Cell^B v3.4 software (Olympus^®^, Münster, Germany). Parasite intensity was assessed as the number of parasites present in at least 3000 erythrocytes (minimum detectable intensity of infection of 1 parasite per 3000 erythrocytes). Samples for which presence of parasites had been identified during the initial perusing but not during the erythrocyte counting were assigned the minimum detectable value (1/3000).

To be able to compare the two methods, we followed the same methodology from the microscopy analyses on a subset of 32 samples from 2011 to 2013. Using R v4.4.3 (R Core Team 2024), the parasite infection intensity values obtained from both methods were fitted to a Generalized Linear Model (GLM) with Gamma distribution and using the log link function, using the values from qPCR intensity as response variable and logarithm of the number of parasites per 3000 erythrocytes (microscopy data) as predictor. The resulting model was used to transform microscopy intensity values into equivalent qPCR intensity values; values equal to 0 in the response variable (qPCR) were forced into 0 on the transformed variable. We used a Bland-Altman plot (package: *blandr*) and a Passing-Bablok regression (package: *mcr*) to assess agreement between observed and transformed values. Most of the data points (93.75%) in the Bland-Altman plot were within ± 1.96 SD of the mean difference and there was no observable bias between the values, suggesting overall agreement; Passing-Bablok regression’s slope (est = 5.82, 95% CI [-10.72, 42.20]) and intercept coefficients (est=-11.67, 95% CI [-97.84, 28.45]) respectively included 1 and 0 in their 95% confidence intervals (CI), suggesting a good agreement and no significant bias between the observed and transformed values. This transformation was then applied to all microscopy intensity values from 2021, with all values equal to 0 prior to the transformation being forced to remain at 0, to obtain a value comparable to the qPCR intensity values from 2011 to 2013.

The final parasite infection parameters used for the statistical analyses were infection status, representing whether or not an individual sample was parasitized and used to directly infer on infection prevalence in the populations, and infection intensity, representing the real and transformed qPCR values (respectively for samples from 2011 to 2013 or 2021) and directly used as a measure of overall infection intensity for the populations.

### Microclimate data

We used NicheMapR (package: *NicheMapR*; Kearney and Porter 2020) to retrieve microclimate data for a representative location of the sampling area (coordinates 41.836, -8.874), using the “micro_ncep” model included in the package. This provides hourly 30 × 30 m gridded microclimatic data, downscaled from 6-hourly 2.5 × 2.5-degree climatic data with account for local terrain effects. We retrieved data as estimated for 1 cm above ground, considering the lizards size, for the period corresponding to the 30 days prior to each sampling day. We then calculated the average, median, maximum, and minimum for the microclimatic variables from NicheMapR. Considering the importance of circadian rhythms for lizards (Dayananda et al. 2020; Vidan et al. 2017), we calculated a second dataset considering separate average, median, maximum, and minimum values for day and night for the variables of air temperature, relative humidity, wind speed, solar radiation, and sky radiant temperature, using the zenith angle of the sun, included in NicheMapR, to differentiate between day and night periods, with night corresponding to any hour with the sun below the horizon.

To facilitate the interpretation of microclimatic effects, we performed principal component analysis (PCA; package:*stats*) on the calculated microclimatic variables average, median, maximum, and minimum values, after centring and scaling the datasets. We selected the number of PCs to retain from each PCA considering the proportion of variance explained by each PC and using the Kaiser criterion (Kaiser 1960), and applied a varimax rotation (Budaev 2010) to the retained PCs (S1 Table), extracting the rotated loadings for analysis. After applying the PCA, we observed that the microclimatic dataset differentiating between day and night values for certain variables did not provide additional information, as most of the day/night variables clustered together with similar effects on the respective PCs (S1 Table), and therefore we discarded this dataset from further analyses.

### Statistical analysis

Parasite infection was assessed as two variables: infection status (parasitized/non-parasitized), and infection intensity. Since, for this specific study system, prevalence is generally low, resulting in a lot of non-parasitized individuals with infection intensity being 0, infection intensity was analysed using a data subset containing only the parasitized individuals.

Using R v4.4.3, a global model was built for each analysis, including all predictor variables (described in detail further ahead). We used an adjusted Variance Inflation Factor (VIF) function (package: *performance*; Lüdecke et al. 2021) to check for multicollinearity within the global model without variable interactions, iteratively dropping the highest scoring variable until all VIF scores were under a maximum threshold of 5 (James et al. 2023; Neter et al. 1996). We performed a Multi-Model Inference with the *dredge* function (package *MuMin*; Bartoń, 2026), using an information criterion corrected for small sample sizes (AICc) to find the most informative models (i.e.: those with AICc scores differing by less than 2 from the best scoring model; Bedrick and Tsai 1994) and used those top models to estimate a final averaged model. We used the conditional model-average coefficients to calculate the relative importance of each predictor in the final average model and retrieve the estimated significance (α < 0.05) of the effects and their z-standardised ß coefficient (Estimate) ± adjusted standard error, to identify the effect direction and strength. We tested the most informative models (without weights, as these are not supported by the *DHARMa* package) simulated residuals for uniformity, quantile deviation, overdispersion, and (for infection status analyses only) zero-inflation (package: *DHARMa*; Hartig et al. 2024).

To assess changes in parasite infection across time and identify the effects of the considered variables, we analysed parasite infection status (parasitized/non-parasitized, representing prevalence when considering the population level) and intensity using Generalized Linear Models (GLMs) with, respectively, binomial and gamma (log link) distributions. For each parasite infection parameter, we performed 3 analyses: (1) identification of temporal trends in parasite infection, with the predictor variables included in the global model being species, sex, SVL, and year and season of sampling; (2) identification of effects of microclimatic variables, with predictor variables in the global models including species, sex,, SVL, year and season of sampling, and the microclimatic variables as the previously estimated PCs, as well as the interactions of species with sex (considering effects of sex could differ between species) and of species and sex with all the other variables (considering species or sexes could have different effects in response to the effects of the other variables); and lastly (3) assessment of the effect of tail condition in parasite infection dynamics, with the global model identical to that of analysis 2, except for the addition of the predictor tail condition. Seasons were defined as the climatic season for Portugal (winter: Dec-Feb, spring: Mar-May, summer: Jun-Aug, autumn: Sep-Nov). Assessment of the effect of tail condition was performed in a separate analysis as this variable had not been recorded in April 2023 and including it in the models implied a loss of a sizeable number of samples (117 samples, 18.4% of sampling pool). Therefore, for both the infection status and infection intensity, analysis 3 was performed on the data subset containing only the samples for which this information was available.

To account for the fact that qPCR detection is a more sensitive method for blood parasite detection than microscopy analyses of blood smears (Maia et al. 2014), we downweighed the importance of the negatives from microscopy in the model by the probability of an obtained negative to be a true negative (the certainty of microscopy negatives), to compensate for the heterogeneity from the methods differing sensitivities and mitigate its influence on the results (Anderson and Bailer 2010; McCullagh and Nelder 1989). To obtain this probability, we divided the expected true negatives by the observed negatives (*n* = 77). Since we know that microscopy detection misses approximately 33% of positives as false negatives in comparison to qPCR (19 false negatives for 38 observed positives according to (Maia et al. 2014), we can calculate the expected true positives by applying the inverse percentage (loss of 33% equates to an increase of 50%) to the observed positives (*n* = 25) and subtract this number from the total samples (*n* = 102) to obtain the expected true negatives. This is summarized with the formula:

$$non-parasitized_{expected true}/non-parasitized_{observed}=$$

$$=(totalsample--parasitized_{observed}\times1.50)/non-parasitized_{observed}$$

which, after replacing with the relevant values, gives a probability of approximately 84% of an observed negative to be a true negative in our sample, which can be applied to the model as a weight of 0.84 to the negatives from microscopy (versus a standard weight of 1.00 for all other observations).

## Results

**Fig. 1:**
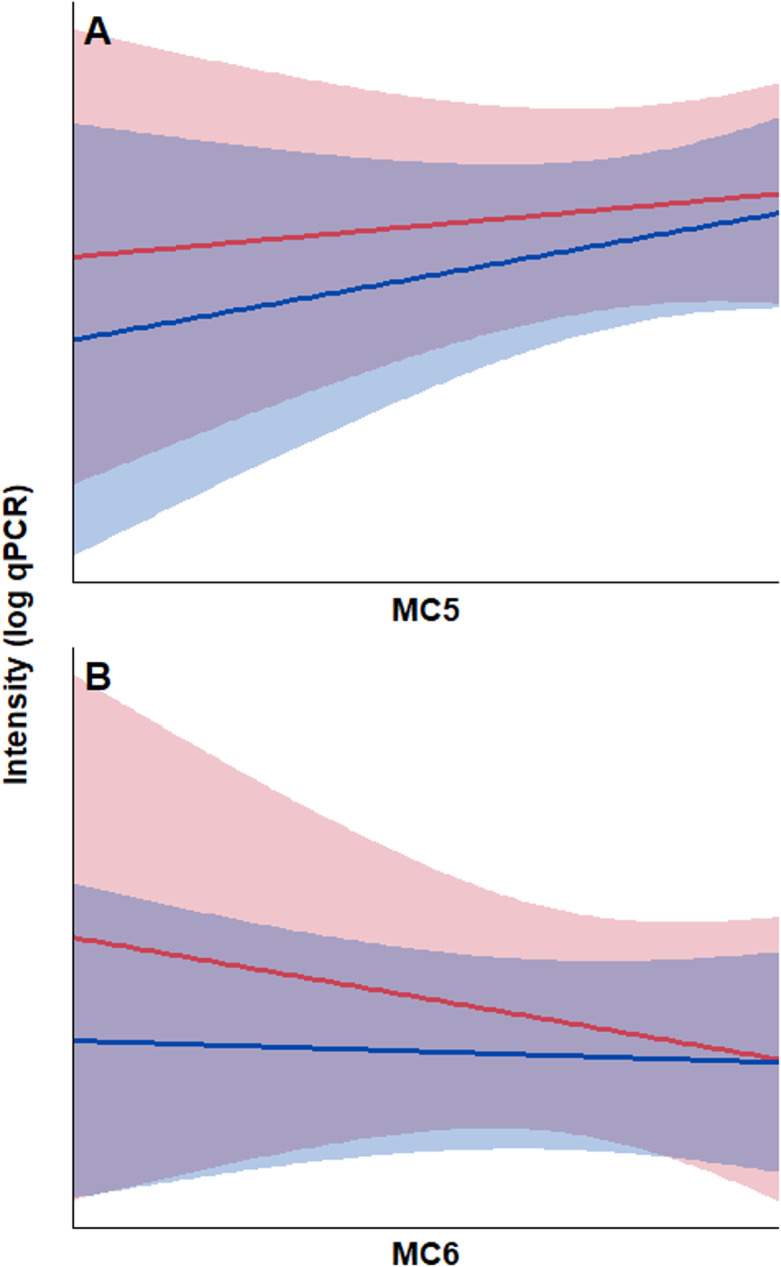
(**A**) Blood parasite prevalence (%) for all sampled individuals and (**B**) intensity (as the logarithm of qPCR number of parasite copies) for parasitized individuals across the 4 sampling years, total values (orange) and discriminated by species (*P. bocagei* in blue and *P. lusitanicus* in green) and sex (males in darker hue and females in lighter hue). Blood parasite intensity values for 2021 are the transformed qPCR number of parasite copies

From the 636 sampled individuals, 393 were parasitized with *Karyolysus* sp. This corresponds to a mean ± standard deviation (SD) prevalence of 62% ± 6% for the years 2011–2013 and a prevalence of 25% for 2021. Parasite infection intensity, within parasitized individuals only, had an overall mean ± SD of 17.42 ± 14.98 and median of 12.60 in qPCR number of parasite copies (measured for 2011–2013 and transformed from microscopy counts for 2021). Yearly numbers of sampled and parasitized individuals, parasite infection prevalence, and median parasite infection intensity can be seen in Table 1; Fig. 1.

**Table 1:** Summary of the number of sampled and parasitized individuals and parasite infection prevalence and intensity (with standard deviation) across sampling years. Intensity considers only parasitized samples and appears as mean ± standard deviation of observed qPCR number of parasites copies for the years 2011–2013 (qPCR), transformed qPCR number of parasites copies for the year 2021 (qPCR-transf), and parasite microscopy counts per 3000 erythrocytes for 2021 (microscopy)

		*P. bocagei*			*P. lusitanicus*			Total
		**Male**	**Female**	**Total**	**Male**	**Female**	**Total**	
2011	**sample size**	92	77	169	64	31	95	264
	**parasitized**	66	46	112	52	19	71	183
	**prevalence**	0.72	0.60	0.66	0.81	0.61	0.75	0.69
	**qPCR**	16.6 ± 11.8	18.9 ± 15.9	17.6 ± 13.6	18.6 ± 11.1	21.7 ± 12.2	19.4 ± 11.4	18.3 ± 12.8
2012	**sample size**	44	29	73	21	11	32	105
	**parasitized**	33	18	51	19	10	29	80
	**prevalence**	0.75	0.62	0.70	0.90	0.91	0.91	0.76
	**qPCR**	14.7 ± 20.3	17.9 ± 18.3	15.8 ± 19.5	21.0 ± 22.6	19.8 ± 24.8	20.6 ± 22.9	17.5 ± 20.8
2013	**sample size**	67	39	106	32	27	59	165
	**parasitized**	45	19	64	21	20	41	105
	**prevalence**	0.67	0.49	0.60	0.66	0.74	0.69	0.64
	**qPCR**	13.4 ± 10.1	16.6 ± 14.3	14.3 ± 11.5	17.5 ± 16.5	16.8 ± 13.4	17.2 ± 14.9	15.5 ± 12.9
2021	**sample size**	36	27	63	25	14	39	102
	**parasitized**	12	3	15	5	5	10	25
	**prevalence**	0.33	0.11	0.24	0.20	0.36	0.26	0.25
	**qPCR-transf**	17.3 ± 15.2	11.3 ± 0.1	16.1 ± 13.7	25.9 ± 18.7	20.5 ± 20.1	23.2 ± 18.6	19.0 ± 15.8
	**microscopy**	1.2 ± 0.2	1.3 ± 10.6	1.3 ± 9.6	14.8 ± 13.5	1.4 ± 14.1	1.95 ± 13.4	1.3 ± 11.4

**Fig. 2:**
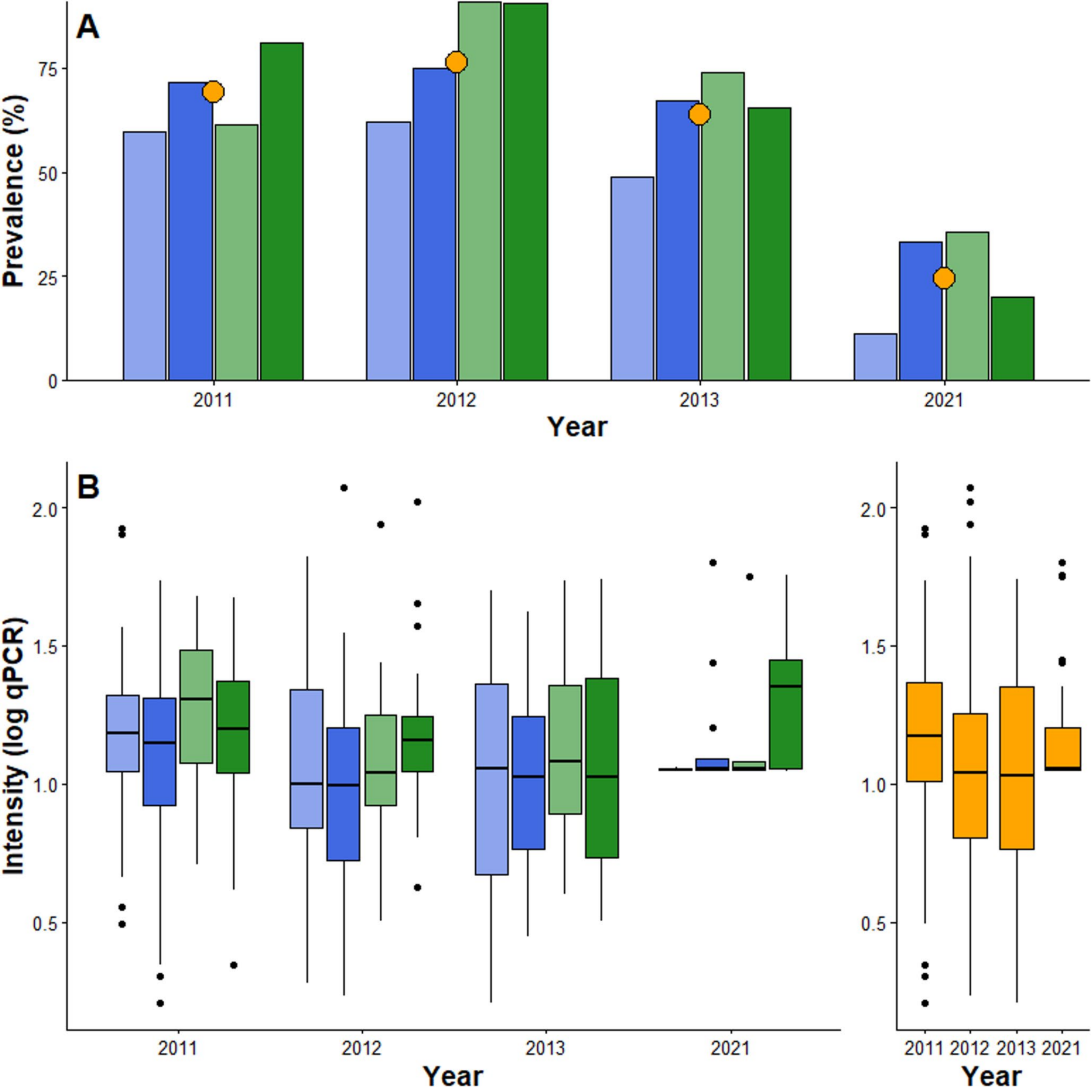
Method comparison and agreement evaluation between observed and transformed qPCR number of parasite copies: (**A**) plot showing the dispersal of observed values (grey) vs. the gamma-shaped distribution of transformed values of qPCR number of parasite copies (orange) for the 21 parasitized samples from 2011–2013 used for model estimation, and (**B**) histogram of the distribution of microscopy counts of all samples from 2021 and (**C**) of the transformed qPCR number of parasite copies for the same samples

For the comparison of the two models, 21 of the 32 random samples from 2011 to 2013 were qPCR positives for *Karyolysus* sp. blood parasites (qPCR: mean ± SD = 16.16 ± 8.06; median = 12.65 min = 11.04; max = 43.04). All those 21 were also positive with the microscopy assessment and were used to build a model to predict qPCR parasite copy numbers from microscopy counts. While the nature of the GLM-obtained distribution for the transformed qPCR parasite copy numbers cannot accurately represent the empiric dispersion between the two methods (Fig. 2A), comparing the histograms of the distribution on the microscopy counts and the corresponding transformed qPCR parasite copy numbers reveals a similar pattern (Fig. 2B), indicating the model can be used to predict this measurement for further analysis. Comparing the transformed qPCR parasite copy numbers with the empirically obtained ones, we observe that the distribution of the transformed values for the 2021 samples (mean ± SD = 18.95 ± 15.84; median = 11.32; min = 11.04; max = 63.13) has a higher minimum and lower maximum than the empirical values from 2011 to 2013 (mean ± SD = 17.42 ± 14.98; median = 12.60, min = 1.60; max = 117.90), but similar mean ± SD and median. This higher minimum is in line with the reduced sensitivity of microscopy blood parasite assessment, resulting in this traditional method missing samples on the lower ranges of parasite intensity.

Temporal assessment of changes in parasite infection status identified significant relationships with year of sampling, with a significant decrease in prevalence in 2021, and an almost significant decrease in 2013, with the variable “year” of maximum importance and present in all top models (Table 2). An almost significant relationship was also identified for sampling season, with samples collected in spring showing a trend for having more parasitized samples, although this variable did not appear in all top models (Importance = 0.40; Table 1). Given the unbalanced sampling distribution across months/seasons, however, results concerning seasonal dynamics should be considered with caution.

**Table 2:** Results of model averaging for effects on parasite infection status, using the full and tail-condition datasets, indicating the number of models selected for creating the final average model (AICc ≤ 2). Significant relationships (*p* ≤ 0.05) are marked in bold. Adj SE = adjusted standard error; SVL = snout-to-vent length. For the interpretation of MC1–MC6 see Table S1

Parasite infection status across time (full dataset; *n* = 636)					
*(6 models)*	Importance	Estimate	Adjusted SE	z value	Pr(>|z|)
(Intercept)		-6.428	0.798	8.051	< 2e-16
Species (*P. lusitanicus*)	1.00	0.449	0.265	1.696	0.090
Sex (male)	0.12	0.107	0.200	0.535	0.593
**SVL**	**1.00**	**0.142**	**0.016**	**8.855**	**< 2e-16**
Year (2012)	1.00	0.181	0.349	0.517	0.605
Year (2013)	1.00	-0.504	0.293	1.724	0.085
**Year** **(2021)**	**1.00**	**-1.916**	**0.411**	**4.666**	**0.000**
Year (2012) : Species (*P. lusitanicus*)	0.31	0.944	0.753	1.254	0.210
Year (2013) : Species (*P. lusitanicus*)	0.31	0.222	0.497	0.447	0.655
Year (2021) : Species (*P. lusitanicus*)	0.31	-0.820	0.601	1.364	0.172
Season (spring)	0.40	0.604	0.345	1.753	0.080
Season (summer)	0.40	-0.044	0.271	0.160	0.873
Season (spring) : Species (*P. lusitanicus*)	0.11	-0.588	0.481	1.220	0.222
Season (summer) : Species (*P. lusitanicus*)	0.11	0.332	0.526	0.631	0.528
Parasite infection status and microclimate (full dataset; *n* = 636)					
*(9 models)*	Importance	Estimate	Adj. SE	z value	Pr(>|z|)
(Intercept)		-6.661	0.808	8.240	< 0.001
**Species (** ***P. lusitanicus*** **)**	**1.00**	**0.506**	**0.215**	**2.350**	**0.019**
Sex (male)	0.07	0.086	0.200	0.429	0.668
**SVL**	**1.00**	**0.142**	**0.016**	**8.845**	**< 0.001**
**MC1**	**1.00**	**-0.137**	**0.063**	**2.175**	**0.030**
MC1 : Species (*P. lusitanicus*)	0.18	-0.103	0.133	0.779	0.436
**MC2**	**1.00**	**-0.157**	**0.039**	**3.993**	**< 0.001**
MC2 : Species (*P. lusitanicus*)	0.74	-0.092	0.061	1.514	0.130
MC4	1.00	-0.111	0.069	1.609	0.108
MC4 : Species (*P. lusitanicus*)	0.35	-0.143	0.120	1.184	0.236
MC5	0.08	-0.022	0.062	0.350	0.726
**MC6**	**1.00**	**-0.392**	**0.079**	**4.974**	**< 0.001**
MC6 : Species (*P. lusitanicus*)	0.08	-0.084	0.140	0.598	0.550
Parasite infection status and microclimate (tail-condition data subset; *n* = 519)					
*(41 models)*	Importance	Estimate	Adjusted SE	z value	Pr(>|z|)
(Intercept)		-7.337	188.318	0.039	0.969
Species (*P. lusitanicus*)	1.00	7.379	424.912	0.017	0.986
Sex (male)	0.57	1.415	248.403	0.006	0.995
Sex (male) : Species (*P. lusitanicus*)	0.54	-0.865	0.472	1.833	0.067
**SVL**	**1.00**	**0.134**	**0.018**	**7.290**	**< 0.001**
SVL : Species (*P. lusitanicus*)	0.02	-0.017	0.039	0.422	0.673
Tail condition (original)	0.60	0.982	242.296	0.004	0.997
Tail condition (regenerated)	0.60	1.390	242.297	0.006	0.995
Tail condition (original) : Sex (male)	0.04	-15.061	929.641	0.016	0.987
Tail condition (regenerated) : Sex (male)	0.04	-15.580	929.641	0.017	0.987
Tail condition (original) : Species (*P. lusitanicus*)	0.43	-15.984	647.897	0.025	0.980
Tail condition (regenerated) : Species (*P. lusitanicus*)	0.43	-15.095	647.897	0.023	0.981
MC1	0.77	-0.118	0.063	1.879	0.060
**MC2**	**1.00**	**-0.171**	**0.046**	**3.686**	**< 0.001**
MC2 : Species (*P. lusitanicus*)	0.77	-0.125	0.073	1.721	0.085
MC3	0.37	-0.105	0.096	1.095	0.274
MC3 : Species (*P. lusitanicus*)	0.10	-0.185	0.130	1.426	0.154
MC3: Sex (male)	0.17	0.175	0.106	1.647	0.100
MC4	0.85	-0.123	0.069	1.774	0.076
MC4 : Species (*P. lusitanicus*)	0.09	-0.109	0.105	1.031	0.303
MC5	0.08	-0.053	0.057	0.935	0.350
MC6	0.02	0.150	0.244	0.614	0.539

For the global model for parasite infection status with the full dataset (*n* = 636) and considering microclimatic variables, VIF analysis indicated extreme correlation and required the removal of sampling season (VIF = 1150.20) and sampling year (VIF = 47.24), before the highest VIF < 5.00 (MC1; VIF = 3.55). In the resulting final averaged model, infection status was significantly related to host species and SVL, with higher prevalence for *P. lusitanicus* and larger lizards overall (Table 2). Relationships with microclimate variables indicated positive significant relationships with maximum wind speed and soil surface wetness (MC1), and with ambient temperature (MC2), and negative significant relationships with relative humidity (MC6; Table 2).

With the data subset including tail condition (*n* = 519), VIF analysis indicated the same highly correlated variables, sampling season (VIF = 1279.67) and sampling year (VIF = 114.69), before the highest VIF < 5.00 (MC1; VIF = 3.94). However, running the resulting model produced a warning of occurrence of fitted probabilities of 0 or 1, indicating the obtained coefficients and p-values from this specific analysis may be unreliable. Analysis of the resulting final averaged model did not identify a significant relationship of infection status with tail condition, or any additional significative relationships other than those with the full dataset, although some (MC1 and MC6) are lost (Table 2).

Temporal assessment of changes in parasite infection intensity did not identify any significant relationships with year or season of sampling, despite an almost significant relationship with the latter, suggesting a trend for higher infection intensity during spring (Table 2). Despite this, the variable season was not present in all top models (Importance = 0.55; Table 2) and, given the unbalanced sampling distribution across months/seasons, this result concerning seasonal dynamics should be considered with caution. Residual analysis of the top models for this analysis failed the dispersion (*p* = 0.035) and Kolmogorov–Smirnov (KS; *p* = 0.016) tests, but considering that visual patterns on the residuals QQ plot appear fine, the variance is close to 0.083 (variance = 0.079), meaning DHARMa residuals behave like a uniform distribution as expected under a well-fit model, and the models passed all other DHARMa tests, the top models fit and dispersion are considered acceptable within the context of noisy parasitological data from wild populations.

**Fig. 3:**
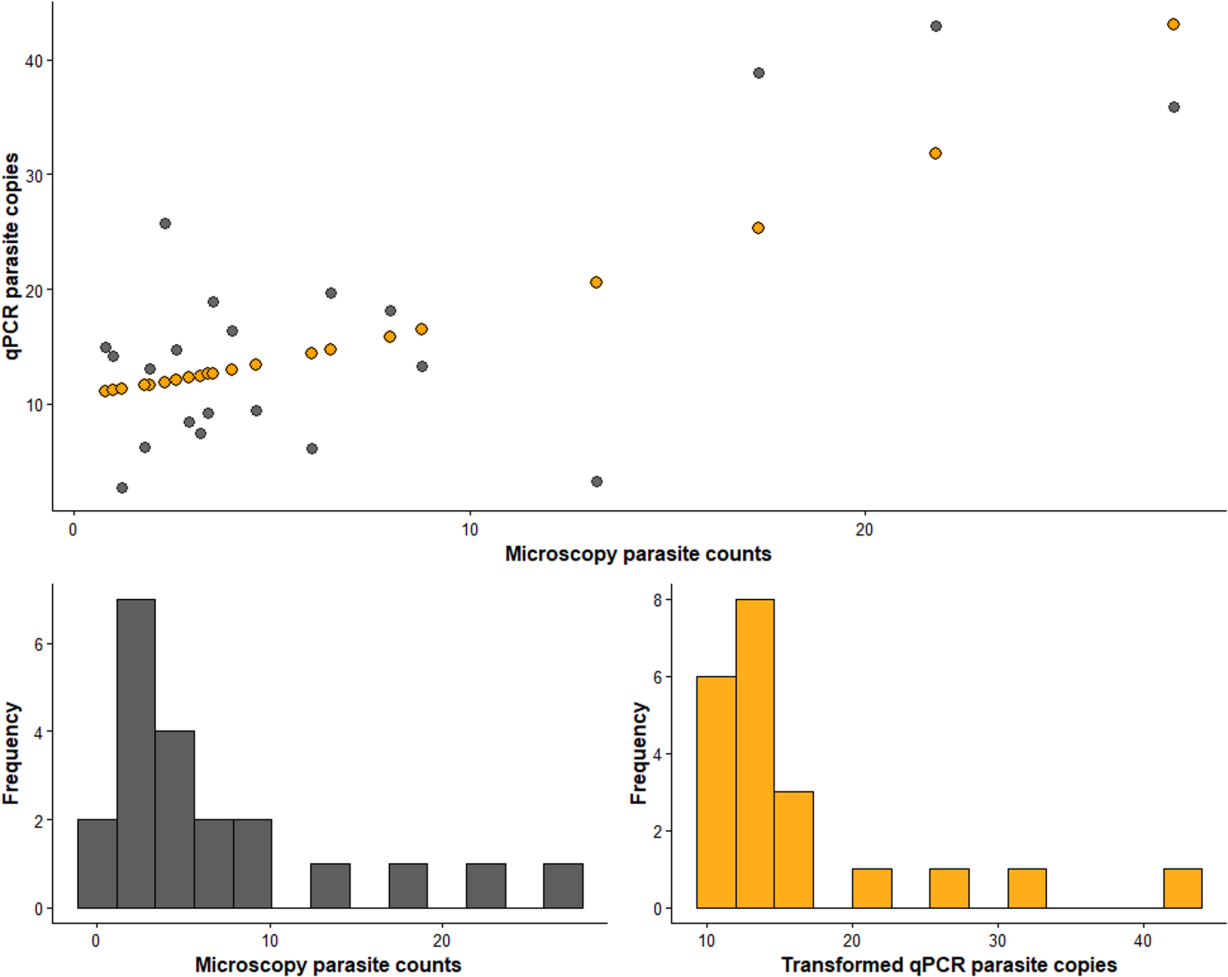
Differential intensity of effects of MC5 and MC6 on the infection intensity (as the logarithm of qPCR number of parasite copies) for males (blue) and females (red) of both *Podarcis* species included in this study

In the global model for parasite infection intensity with the full dataset of parasitized samples (*n* = 393) and considering microclimatic variables, VIF analysis required removing sampling season (VIF = 972.65) and sampling year (VIF = 31.74), before the highest VIF < 5.00 (MC1; VIF = 2.77). In the final averaged model, infection intensity was significantly related to host species, with *P. lusitanicus* hosting more intense infections than *P. bocagei* (Table 2). Concerning microclimatic variables, only two appear in the top models (MC5 and MC6) but, despite both appearing in all of them (Importance = 1.00), only one is significantly related to overall infection intensity, indicating infection intensity increased with lower relative humidity (MC6; Table 2). However, significant interactions between sex and these two microclimatic variables were also identified, indicating that the significant effect of relative humidity (MC6) differs between males and females of both species (Table 3), with the effect being significantly stronger for females (Fig. 3B). Similarly, although water pooling on surface and dew formation (MC5) did not have a significant effect on overall infection intensity for the two lizard populations, a decrease in these variables had a positive effect on infection intensity, that was significantly stronger on females than males of both species (Table 3; Fig. 3A). Residual analysis of the top models again failed the dispersion (*p* = 0.018) and KS (*p* = 0.008) tests but considering the same above-mentioned reasons and context (variance = 0.080), the obtained top models fit and dispersion are considered acceptable.

**Table 3:** Results of model averaging for effects on parasite infection intensity, using only the parasitized individuals from the full and tail-condition datasets, indicating the number of models selected for creating the final average model (AICc ≤ 2). Significant relationships (*p* ≤ 0.05) are marked in bold. Adj SE = adjusted standard error; SVL = snout-to-vent length. For the interpretation of MC1–MC6 see Table S1

Parasite infection intensity across time (full dataset; *n* = 393)					
*(5 models)*	Importance	Estimate	Adjusted SE	z value	Pr(>|z|)
(Intercept)		2.784	0.155	17.956	< 0.001
Species (*P. lusitanicus*)	1.00	0.170	0.090	1.887	0.059
Sex (male)	0.42	-0.100	0.091	1.093	0.274
SVL	0.09	-0.002	0.008	0.220	0.826
Season (spring)	0.55	0.175	0.103	1.688	0.091
Season (summer)	0.55	0.069	0.109	0.635	0.525
Parasite infection intensity and microclimate (full dataset; *n* = 393)					
*(3 models)*	Importance	Estimate	Adjusted SE	z value	Pr(>|z|)
(Intercept)		2.789	0.083	33.759	< 0.001
**Species** **(***P. lusitanicus***)**	**1.00**	**0.188**	**0.088**	**2.123**	**0.034**
Sex (male)	1.00	-0.049	0.093	0.532	0.595
MC5	1.00	-0.014	0.030	0.482	0.630
MC5 : Species (*P. lusitanicus*)	0.23	-0.019	0.035	0.552	0.581
**MC5** **: Sex (male)**	**1.00**	**0.080**	**0.037**	**2.151**	**0.032**
**MC6**	**1.00**	**-0.102**	**0.047**	**2.187**	**0.029**
MC6 : Species (*P. lusitanicus*)	0.24	0.033	0.051	0.635	0.525
**MC6** **: Sex (male)**	**1.00**	**0.116**	**0.057**	**2.053**	**0.040**
Parasite infection intensity and microclimate (tail-condition data subset; *n* = 300)					
*(7 models)*	Importance	Estimate	Adjusted SE	z value	Pr(>|z|)
(Intercept)		2.871	0.344	8.346	< 0.001
Species (*P. lusitanicus*)	1.00	0.103	0.423	0.243	0.808
Sex (male)	1.00	0.005	0.111	0.043	0.966
SVL	0.11	-0.271	0.376	0.721	0.471
Tail (original)	0.11	1.087	0.644	1.687	0.092
Tail (regenerated)	0.11	1.219	0.631	1.932	0.053
Tail (original) : Species (*P. lusitanicus*)	0.26	-0.009	0.009	0.909	0.363
Tail (regenerated) : Species (*P. lusitanicus*)	0.11	-0.104	0.383	0.272	0.786
MC5	1.00	0.003	0.031	0.082	0.935
MC5 : Sex (male)	0.75	0.069	0.037	1.857	0.063
MC5 : Species (*P. lusitanicus*)	0.05	-0.010	0.038	0.279	0.780
MC6	0.11	-0.038	0.086	0.447	0.655

With the data subset including only parasitized samples with tail condition information available (*n* = 300), VIF analysis still identified extreme correlation for sampling season (VIF = 603.42) and sampling year (VIF = 70.93), before the highest VIF < 5.00 (MC1; VIF = 3.51). No significant relationships were identified, despite the presence of some almost significant trends related to tail condition, indicating individuals with regenerated tails (*n* = 182) boasted the higher infection intensity and the ones with lost tails (*n* = 9) the lowest, while those with original tails (*n* = 109) were somewhere in between (Table 3). While the difference between original and regenerated tails is plausible according to the literature (Oppliger and Clobert 1997) the result on lost tails should be considered cautiously, given the low number of individuals in this condition. Residual analysis of the top models also failed the dispersion test (*p* = 0.032), but these were still considered acceptable given remaining tests (variance = 0.081) and context of data.

## Discussion

This 10-year apart assessment of blood parasite infection by the genus *Karyolysus* in this lizard community offers new insights into the temporal dynamics of this infection. Our results indicate that significant relationships between blood parasite infection and individual lizard traits stay consistent across years and climatic variation. From the analysis on the individual parameter of parasite infection status, we can infer that parasite infection prevalence, at a population level, trended down within the sampled years, with this drop being significant in 2021 in relation to the remaining years, even after accounting for a lesser sensitivity from the detection method. Parasite infection intensity, on the other hand, did not have any detectable significant seasonal or yearly trends. Analysis of the larger dataset indicated prevalence was significantly higher for *P. lusitanicus*, and across larger individuals, but significant relationships with infection intensity were harder to ascertain as analysis of both datasets proved equally reliable but produced different results. There was a significant relationship for infection intensity with host species, using the larger dataset of parasitized individuals, indicating that *P. lusitanicus* individuals host higher parasite infections than *P. bocagei*. While this relationship was not significant in the analysis of the smaller dataset of parasitized individuals (including tail condition data), host species still appears in both analysis as a predictor of highest importance for infection intensity, being present in all the top models used for both final averaged models (Importance 1.00; Table 3). Infection intensity also had an almost significant relationship with tail condition in the smaller dataset, but this predictor had very low importance in that analysis (Importance = 0.11, Table 3). Microclimatic variables identified as significant for infection prevalence, indicated overall warmer and drier conditions lead to higher prevalence, despite a confounding effect from increased wind speed and soil surface wetness. The latter, while usually linked to lower temperatures and more humid conditions, also had a significant positive relationship with infection prevalence. Similarly to parasite infection prevalence, infection intensity also significantly increased with drier conditions. However, the magnitude of its responses to microclimatic conditions varied significantly according to host sex, despite always maintaining the same direction. The difference in significant relationships for parasite infection prevalence and intensity suggest infection transmission and growth respond differently to host traits and external changes, which would explain why the observed large decrease in infection prevalence was not accompanied by an equivalent decrease in infection intensity.

While our analyses identify a positive significant relationship between parasite infection prevalence and warmer, drier climatic periods, for the study years of 2011–2013 and 2021, Moledo registered a cooling pattern, with 2011 having the highest positive mean annual temperature anomaly of the four years, 2012–2013 having negative anomalies, and 2021 having a very low positive anomaly (IPMA, 2025). This could explain the observed decrease in *Karyolysus* parasite infection parameters in these lizard host population across the sampled years and suggest a trend for decreasing infection by blood parasites in these *Podarcis* lizard host populations. However, the lack of intermediate time points means this downward trend cannot be fully ascertained. Additionally, between 2013 and 2021, there were 3 years of positive climatic anomalies greater than that of 2011 (2015, 2017, and 2020) and only one negative climatic anomaly was registered (2018). The year 2021 was also followed by 3 years of positive mean annual temperature anomalies all higher than that of 2011. All this indicates the actual regional climatic trend is towards a mean annual temperature increase (IPMA, 2025) and, considering the observed positive significant relationship between infection prevalence and ambient temperature, would suggest an expected trend of increasing infection prevalence, with 2021 possibly corresponding to an isolated, out-of-trend drop due to its very low positive anomaly (IPMA, 2025). Smaller, partial parasite assessments from other studies in the same locality recovered similar, although still higher, values of *Karyolysus* prevalence to those of 2021 (25%), with 36% in 2022 (39 males from both species; Faria et al. 2024) and 29% in 2023 (28 males of *P. lusitanicus*; Faria 2025). This suggests that the infection prevalence observed for 2021 is not the result of an isolated drop but also hints at a possible increase in infection prevalence together with a rise of ambient temperatures in the following years (IPMA 2025), concurrent with the observed positive significant relationship between infection prevalence and warmer, drier conditions. However, we cannot confirm this, due to the partial status of the data available for the following years (missing females in both years and one of the two species in 2023).

In line with our results, other studies considering the effect of climate on endoparasite infection in lizard hosts commonly observe a positive correlation of blood parasite infection prevalence and intensity with higher temperatures and lower humidity (Álvarez-Ruiz et al. 2018; Drechsler et al. 2021; Huyghe et al. 2010; Megía-Palma et al. 2024), but also with environmental stress caused by lower (Bower et al. 2019; Oppliger et al. 1998) or unstable temperatures (Paranjpe et al. 2014). Invertebrate ectoparasites (vectors of blood parasites) are also impacted by climate (Paranjpe et al. 2014; Zamora-Vilchis et al. 2012), with hematophagous mites (order Mesostigmata) and ticks (order Ixodida), the putative vectors for *Karyolysus* sp. in Iberian lizards, increasing their prevalence and activity with higher temperatures (Drechsler et al. 2021; Pollock et al. 2015; Rulison et al. 2014), but also at higher altitudes, usually associated with lower temperatures (Álvarez-Ruiz et al. 2018; Wu et al. 2019), although a case can be made in higher altitudes for the relationship to be with greater seasonal temperature variation, instead of a clear decrease in it. These effects in invertebrate vectors could possibly alter the intensity of contact with vertebrate hosts and affect blood parasite transmission.

Increased wind speed and soil surface wetness were also significantly related to increased parasite infection prevalence. While these would generally result in lower effective ambient temperatures and increased humidity, indicating a relationship contrary to that of the other significant microclimatic variables, they had a slightly weaker effect on parasite infection prevalence (MC1 Estimate = -0.137 ± 0.063) than the other two (MC2 Estimate = -0.157 ± 0.039 and MC6 Estimate = -0.392 ± 0.079), suggesting ambient temperature and relative humidity would remain the main microclimatic predictors of infection prevalence. Furthermore, the effects of wind speed and soil surface wetness could be not related to how they affect effective ambient temperature and the subsequent impact on lizard vulnerability to parasite infection, but on how it directly affects lizard behaviour. It could be that during periods of increased wind speed and higher soil surface wetness, *Podarcis* populations spend less time basking and foraging and more time in in groups hiding inside refugia (Ortega and Pérez-Mellado 2016), increasing their chances of exposure to vectors and of contracting blood parasites from other individuals. Furthermore, these conditions may also be linked to increased environmental stress, with immunosuppressive effects that may render the lizards more vulnerable to the establishing of parasite infections they might come into contact during these periods (Hussein et al. 1979; Innis et al. 2009; Oppliger et al. 1998).

Blood parasite infection intensity was not significantly related to any microclimatic variables. The different relationships of prevalence and intensity with microclimatic variables indicate that the mechanisms controlling the two variables are different (Ferreira et al. 2023). One possible explanation would be how prevalence is more closely linked to vectors (Amo, López, et al. 2005; but see also Schall and Marghoob 1995), and therefore also affected by microclimatic changes also affecting these organisms, while intensity should be more independent from vectors and subject to specific responses of the lizard host and blood parasite only.

Differences in parasite infection for closely phylogenetically related hosts can usually be explained by differences in both the host organisms themselves and the habitat each are present in (Eisen and Wright 2001; Hawley and Altizer 2011). Considering the studied lizard populations share the same macro-habitat and are exposed to very similar microclimatic conditions, interspecific differences in prevalence and intensity are likely explained by host organism differences. The two host species present in this study have differential microhabitat usage (Carretero et al. 2002; Kaliontzopoulou et al. 2012), which in turn could influence vector exposure (Bernal et al. 2007; Kiesecker and Skelly 2000). Studies have identified a positive correlation between vegetation usage and mite load in lacertids (Curtis and Baird 2008), however *P. bocagei* is the species considered more generalist while *P. lusitanicus* is more of a rock-specialist (Kaliontzopoulou et al. 2012), which would go against that correlation as we would expect *P. bocagei* would have higher contact with vegetation, indicating factors other than type of microhabitat-usage influence this interspecific difference. It also appears unlikely that the reduced prevalence in *P. bocagei* would be due to the adaptation of behaviours by this species to minimise parasitical infection, as these cases are rarely observed in reptiles (Bower et al. 2019). Individuals of *P. lusitanicus* parasitized with *Karyolysus* sp. were observed to move more, and more erratically compared to uninfected individuals (Faria et al. 2024), which, despite being already post-establishment of infection for those specific individuals, could further increase the species exposure to vectors as the parasitized individuals may carry the vectors further into contact with other members of the species, especially in circumstances of higher population density (Megía-Palma et al. 2024). These results are similar to those obtained for another location in Northern Portugal (Gerês; 41.782, -8.145) where these species also occur in sympatry, where *P. lusitanicus* also displayed higher prevalence compared to *P. bocagei* (89% vs. 73%), and where again there was no significant relationship between intensity and host species (Maia et al. 2014). When it comes to infection intensity, interspecific physiological and immune responses to the parasite might therefore play a role (Faria 2025), despite the overall ecological similarity and close phylogenetic relationship between *P. lusitanicus* and *P. bocagei*.

Within a species, sex is a well-known factor for differential parasite infection and response. For parasite infection intensity, certain microclimatic variables had significantly different intensity of effect between the host sexes, in both cases more intense for females. This would suggest significant variation in the physiological responses of the hosts to microclimatic conditions can differently affect the vulnerability of each sex (Sacchi et al. 2017), although the lack of an observed immune response in this genus when infected by blood parasites (Damas Moreira et al. 2022) means it is unclear which factors could be differently affected for each sex by microclimatic conditions.

Variation in endoparasite infection due to body size and condition have already been widely reported before across lizard taxa, with the generally accepted hypothesis that larger individuals are more likely to be parasitized and boast higher infection intensities than smaller individuals (Amo et al. 2004; Amo, Fargallo, et al. 2005). The mechanism underlying this higher exposure, however, remains uncertain; it could be age-related, with bigger and older individuals having had more chances to contract parasites and more time for the infection to establish and develop throughout their lifespan or simply because of the larger body surface area being easier to target by vectors (Watkins and Blouin-Demers 2019). Furthermore, this relationship is not always observed (Damas-Moreira et al. 2014; Er-Rguibi et al. 2021; Garrido and Pérez-Mellado 2013), and even negative relationships have been identified (Smallridge and Bull 2000). In this study, we also observe a significant relationship between larger body size and parasite infection prevalence. The fact that relationships between parasite infection parameters and body measurements are not observed in studies with generally smaller sampling sizes (Damas-Moreira et al. 2014), including from this same population (Faria et al. 2024) might suggest these relationships, even when significant, do not explain a major portion of the variance in parasite infection parameters, making it hard to identify without extensive sampling.

**Fig. 4:**
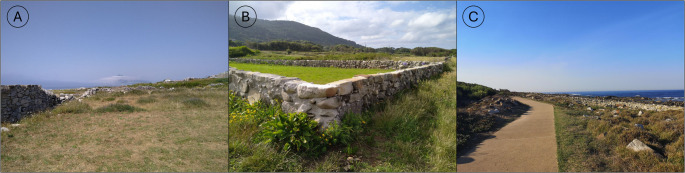
Photographs from (**A**) July 2022 and (**B**) June 2024, showing the encroaching of the urban area towards the rural patches by replacing the traditional land use with mowed grass lawns and the dry-stone walls with cemented ones. (**C**) Section of the paved path of the Ecovia Litoral Norte

Although we did identify a positive significant relationship between *Karyolysus* infection prevalence in *Podarcis* and warmer, drier microclimatic conditions, likely explaining at least a portion of the decline in prevalence between 2011 and 2013 and 2021 as the temperatures decreased in those years, other untested factors might also have contributed to this decrease. During regular assessments of these *Podarcis* populations over the last two decades, it has been notable that the landscape has been progressively changed by anthropogenic action, with an encroachment of the urban area towards the rural and overgrown patches used by the lizards (Fig. 4A, B). In 2017, a new section of a walking and cycling path along the northern coast of Portugal was opened to the public, installing an obstacle for lizard movement in the form of paved path (Fig. 4C) between the walled patches and adjacent habitats that also attracts tourists to the region year-round. Anecdotal reports from other researchers sampling in the same region also point to a concern with the increased difficulty in observing and capturing *Podarcis* sp., suggesting possible population declines. Although habitat fragmentation (Gillespie and Chapman 2008; Perrin et al. 2023) and increased touristic activity (Amo et al. 2006) are usually linked to increases in parasite prevalence, its effects appear to be taxon-specific (Perrin et al. 2023). The reduction of vegetal overgrowth and the increasing habitat fragmentation with a population decline might, respectively reduce the lizards’ exposure to invertebrate vectors and hinder the transmission of these vectors between subpopulations, further accentuating the decrease in prevalence of these blood parasites. The presence of livestock in the area might also influence blood parasite dynamics, with studies showing that livestock is associated with decreases in mite and increases in tick infestation in lizards (Pafilis et al. 2013; Wu et al. 2019). A reduction in sheep and goat presence in the area, due to urban encroachment, could change the densities in the invertebrate vectors and further hinder the transmission and completion of the lifecycle for the *Karyolysus* sp. blood parasite. However, these anthropogenic influences were not measured in our study, and therefore we present them as untested hypotheses and cannot determine whether they have a significant impact in the parasite infection dynamics.

Assessing the current state of blood parasite infection in nearby populations from these species for which previous data is available (e.g., Roca and Galdón 2010), subject to similar microclimatic changes, could provide additional insights into the large decrease in prevalence we observed for our study populations, as it would indicate whether this is a local or more overreaching phenomena. This combination of past and present data is a powerful tool to test specific hypotheses for the future, as we can predict changes to parasite infection parameters based on the climatic conditions registered during recent years and then test if they occur within those predictions in the near future. Furthermore, accounting for the inherent methodological differences (e.g., sensitivity of qPCR vs. microscopy) in the statistical analyses, we can rely on historical data even if collected with different methodologies, broadening the possibilities for this kind of studies.

To our knowledge this is the first study to analyse the dynamics of blood parasite infection in reptilian population with such a wide timespan in the Iberian Peninsula, providing new insights into the maintenance of parasitic relations across time. The extremely high correlation of sampling season and year with the remaining predictors and the fact that microclimatic variables were the only non-host-related predictors included, suggest changes to microclimatic conditions accurately represent the environmental variation across the studied years and reinforce the importance of the impact of climatic changes on parasite infection prevalence. From these, we identified a significant relationship indicating warmer, drier condition increase the prevalence of *Karyolysus* sp. blood parasites in these species, and that drier conditions might also lead to increased infection intensity, with different magnitudes of effect according to host sex. Under a scenario of continued global warming, higher temperatures would benefit these blood parasites (Megía-Palma et al. 2024), and possible lead to an increase of the infection parameters in the region, with potential unbalancing effects on the host populations (Dougherty et al. 2016; Lyles and Dobson 1993; Parker 1971). If this pattern is applicable to other lizard host-parasite pairs around the globe, sudden changes in parasite populations could endanger the balance of many ecosystems in which these organisms play a critical role (Marick et al. 2023; Wood and Johnson 2015). 

## Supplementary Information

Below is the link to the electronic supplementary material.

Supplementary Material 1 (DOCX 29.7 KB)

Supplementary Material 2 (CSV 92.2 KB)

## Data Availability

The data collected and used for the purpose of this research is available as supplementary material to this publication.
