# Supplementary material for: A ten-year change in blood parasite infection in a sympatric wall-lizard community (genus Podarcis) from the Atlantic coast of Portugal
Source: Parasitol Res. 2026 May 18;125(1):81. doi: 10.1007/s00436-026-08693-3 (PMC13346367; doi:10.1007/s00436-026-08693-3)
Supplement: Supplementary file 1 — Supplementary Material 1 (DOCX 29.7 KB) [file 436_2026_8693_MOESM1_ESM.docx]

**S1 Table.** Importance of components and varimax rotation loadings from the principal component analyses (PCAs) performed on the microclimatic variables from NicheMapR. Eigenvalues > 1, used to select the number of principal components (PC) to retain, named MC1-6, are indicated in bold. Rotation loadings are shaded in a gradient from green (positive) to red (negative), with colour intensity matching the increased loading weight in either direction.

| **Importance of components** | | | | | | | | |
| --- | --- | --- | --- | --- | --- | --- | --- | --- |
|  | **MC1** | **MC2** | **MC3** | **MC4** | **MC5** | **MC6** | **MC7** | **MC8** |
| **Standard deviation** | 3.84 | 3.27 | 2.65 | 1.57 | 1.45 | 1.01 | 0.95 | 0.00 |
| **Proportion of Variance** | 0.38 | 0.27 | 0.18 | 0.06 | 0.05 | 0.03 | 0.02 | 0.00 |
| **Cumulative Proportion** | 0.38 | 0.65 | 0.83 | 0.90 | 0.95 | 0.98 | 1.00 | 1.00 |
| **Eigenvalues** | **14.78** | **10.71** | **7.05** | **2.47** | **2.09** | **1.01** | 0.90 | 0.00 |

| **Varimax rotation loadings** | | | |  |  |  |
| --- | --- | --- | --- | --- | --- | --- |
|  | **MC1** | **MC2** | **MC3** | **MC4** | **MC5** | **MC6** |
| **TALOC.avg** | 0.11 | -0.25 |  |  |  |  |
| **TALOC.med** |  | -0.28 |  |  |  |  |
| **TALOC.max** |  |  |  | -0.47 |  |  |
| **TALOC.min** |  | -0.26 |  |  |  |  |
| **TAREF.avg** |  | -0.29 |  |  |  |  |
| **TAREF.med** |  | -0.29 |  |  |  |  |
| **TAREF.max** | -0.12 | -0.24 |  | -0.29 |  |  |
| **TAREF.min** |  | -0.27 |  |  |  |  |
| **TSKYC.avg** |  | -0.27 |  | 0.14 |  |  |
| **TSKYC.med** |  | -0.24 |  | 0.14 |  | 0.17 |
| **TSKYC.max** | -0.19 | -0.31 |  |  |  | -0.20 |
| **TSKYC.min** |  | -0.23 |  |  |  |  |
| **SOLR.avgD** | 0.19 |  |  | -0.18 | 0.10 |  |
| **SOLR.medD** | 0.18 |  |  | -0.19 |  |  |
| **SOLR.maxD** |  |  | -0.21 | -0.15 | 0.17 |  |
| **VLOC.avg** |  |  | -0.36 |  |  |  |
| **VLOC.med** |  |  | -0.36 |  |  | 0.11 |
| **VLOC.max** | -0.45 |  |  |  | 0.13 |  |
| **VLOC.min** | 0.11 |  | -0.39 | 0.22 |  | -0.21 |
| **VREF.avg** |  |  | -0.36 |  |  |  |
| **VREF.med** |  |  | -0.36 |  |  | 0.11 |
| **VREF.max** | -0.45 |  |  |  | 0.13 |  |
| **VREF.min** | 0.11 |  | -0.39 | 0.22 |  | -0.21 |
| **RHLOC.avg** | -0.21 |  |  | 0.13 | -0.10 |  |
| **RHLOC.med** | -0.16 |  |  | 0.17 |  |  |
| **RHLOC.max** |  |  | -0.11 |  |  | 0.55 |
| **RHLOC.min** |  |  |  | 0.46 |  |  |
| **RH.avg** |  | 0.19 |  | 0.15 | -0.15 | 0.20 |
| **RH.med** |  | 0.18 |  | 0.14 | -0.17 | 0.13 |
| **RH.max** |  |  | -0.15 |  |  | 0.50 |
| **RH.min** |  | 0.20 |  | 0.21 | 0.33 | 0.20 |
| **POOLDEP.avg** |  |  |  |  | -0.39 |  |
| **POOLDEP.max** |  |  |  |  | -0.39 |  |
| **PCTWET.avg** | -0.31 | 0.11 |  |  |  |  |
| **PCTWET.med** | -0.34 | 0.16 |  | -0.20 |  | -0.12 |
| **PCTWET.max** | -0.31 | 0.12 | -0.21 | -0.15 |  | -0.32 |
| **PCTWET.min** |  |  |  |  | -0.39 |  |
| **DEW.avg** |  |  |  |  | -0.41 |  |
| **DEW.max** |  |  | -0.17 | -0.12 | -0.32 |  |
